# Mechanical, Adhesive and Surface Properties of a Zirconia-Reinforced Lithium Silicate CAD/CAM Ceramic Exposed to Different Etching Protocols

**DOI:** 10.3390/ma17205039

**Published:** 2024-10-15

**Authors:** Fabián Murillo-Gómez, José Roberto Hernández-Víquez, José Roberto Sauma-Montes de Oca, Cristina Vargas-Vargas, Natalia González-Vargas, José Roberto Vega-Baudrit, Daniel Chavarría-Bolaños

**Affiliations:** 1 Restorative Dentistry Department, School of Dentistry-FOd, University of Costa Rica-UCR, San José 11502, Costa Rica; 2 Dental Materials Research Laboratory (LIMD), School of Dentistry-FOd, University of Costa Rica-UCR, San José 11502, Costa Rica; 3 Dentistry Graduate Program, University of Costa Rica-UCR, San José 11502, Costa Rica; jose.saumamontesdeoca@ucr.ac.cr (J.R.S.-M.d.O.); daniel.chavarria@ucr.ac.cr (D.C.-B.); 4 National Laboratory of Nanotechnology-LANOTEC, National Centre of High Technology-CENAT, San José 10109, Costa Rica; jvegab@gmail.com

**Keywords:** zirconia-reinforced lithium silicate, CAD/CAM materials, glass-ceramics, flexural strength, surface treatment, hydrofluoric acid

## Abstract

The aim of this investigation was to evaluate the effect of etching protocols on bond strength, surface roughness, and mechanical properties of a zirconia-reinforced lithium silicate (ZLS) CAD/CAM-ceramic. In total, 100 bars (ISO 6872), 75 plaques, and 25 cubes were cut from ZLS blocks(Vita Suprinity^®^). The surfaces were standardized, crystallized and divided into five groups: 1. control (no/treatment-C), 2. 5%-Hydrofluoric-acid (HF)/20 s (HF5%20s), 3.HF5%60s, 4.HF10%20s, and 5.HF10%60s. Flexural strength (FS) (three-point bending test, 1 mm/min), roughness (Pa), and micro-shear bond-strength (µSBS) tests were performed. The data were statistically analyzed with one-way ANOVA, Tukey’s test (*p* ˂ 0.05) and Weibull (FS data). C showed higher Pa (1.176 ± 0.370 µm) than HF10%60s (0.627 ± 0.236 µm) and all other groups. Groups C and 20 s showed the most irregular surface patterns. The FS results were not influenced by etching protocols, while the Weibull modulus was, with the 5%HF groups being the most reliable (m: 5.63/6.70), while C and HF10%60s (m: 2.78/2.73) were the least reliable. All fractures originated from surface defects on the treated side of specimens. The 5%HF groups showed higher µSBS (20 s: 21.35 ± 4.70 MPa; 60 s: 23.50 ± 4.27 MPa) than the 10%HF groups (20 s: 14.51 ± 2.47 MPa; 60 s: 16.54 ± 3.12 MPa) and C (6.46 ± 2.71 MPa). The most prevalent failure pattern was “mixed” for etched groups, and “adhesive” for C. Etching protocols affect the evaluated properties by roughening materials’ surface and, in some cases, regularizing surface defects. The best overall outcomes were achieved when applying 5%HF.

## 1. Introduction

Ceramic indirect dental restorations have been popular for many years now; however, their production process has faced significant improvements recently owing to fast growth in automated CAD/CAM technologies. It has revolutionized the traditional laboratory processes, turning them into faster, more reliable, profitable, and controlled procedures, allowing the clinician and patient to obtain high-quality products while reducing chair time and improving treatment convenience [1,2].

This technological evolution has also influenced the dental materials’ development industry, boosting the release of new materials directed specifically to CAD/CAM technologies. Such materials show, in some cases, better properties than their analog versions [3,4,5]. CAD/CAM materials comprise glass-containing ceramics, polycrystalline ceramics, polymers, and hybrid materials [3,6]. These options give the clinician several possibilities of matching materials’ properties with the most appropriate clinical use.

Historically, the industry has tried to provide materials to fulfill mainly two aspects: suitable mechanical properties and proper aesthetical capabilities. One of the best candidates to achieve an ideal balance between these two aspects is lithium disilicate-reinforced ceramic. This material combines a translucent glass matrix with strong crystals, providing outstanding aesthetics and high mechanical properties [4,7]. As this combination of glassy and crystalline-reinforcing phases has proven successful, manufacturers have recently developed similar proposals following this approach [8].

The zirconia-reinforced lithium silicate glass-ceramic (ZLS) was developed as an alternative to lithium disilicate ceramic. Given the excellent results reported in the literature regarding its balanced combination of aesthetics and mechanical properties, and the fact that this material was patented for several years, other manufacturers sought to use lithium silicate technology to create a new material. In this case, Dentsply and VITA developed ZLS using lithium silicate crystalline technology (along with other crystalline structures) and increased zirconia content, aiming to combine the advantages of lithium silicate ceramics with the well-known superior mechanical properties of zirconia-based ceramics. It consists of lithium metasilicate and lithium orthophosphate crystals inserted in a zirconia-powder-added glassy phase [3,9,10]. This material is available in two commercial presentations: press-ingots and CAD/CAM blocks [6]. Its overall properties and behavior are like those of lithium disilicate [3,4,5,8,11,12,13,14,15,16]. However, some adverse outcomes related to its microstructure, Weibull modulus, fatigue lifetime predictions, and fracture behavior have been reported [4,5,15,17,18,19,20,21].

As ceramic bonding with resin cement and tooth tissues is one of the most important factors to ensure long-lasting restorations, the adhesion capabilities of ZLS have also been tested [22,23,24,25]. The suggested surface treatment is to apply a hydrofluoric acid (HF) followed by a silane-containing primer, same as for other glass-ceramics [24,25,26,27,28,29,30,31,32,33,34]. Both strategies work synergistically. Hydrofluoric acid (HF) dissolves part of the glassy matrix in glass-containing ceramics, increasing the surface energy of the material, while the silane acts as a bridge between silica-based ceramics and resin-based materials. Although HF has raised concerns due to its potential toxicity to soft tissues, despite some alternative approaches, it remains the gold standard for improving glass-ceramic bonding when used alongside a silane primer [22,23,24,25]. However, there are still questions about the most suitable etching parameters (concentration/application time) due to significant variability in the studies’ methods and observed outcomes [23,25]. Although ceramic surface treatment’s primary goal is to ensure a reliable bonding of the indirect restoration, concern about its effect on materials’ mechanical properties has been recurrent. This concern has led researchers to investigate the influence of different HF concentrations and application times on the physical–mechanical behavior of glass-ceramics as previously studied [7,30,35,36,37,38,39,40,41,42,43,44,45,46,47,48,49,50,51].

Only a few studies have investigated the influence of HF etching on the mechanical properties of a ZLS ceramic [38,45,49]. Moreover, none have yet evaluated this issue, to the authors’ knowledge, related to other essential properties of this novel material such as roughness and bond strength. Thus, this study aimed to evaluate the effect of different etching protocols on the bond strength, surface roughness and morphology, flexural strength, and Weibull parameters of a zirconia-reinforced lithium silicate CAD/CAM ceramic material. The null hypothesis set was that the etching protocol does not influence the bond strength, surface roughness, flexural strength, and Weibull parameters of a zirconia-reinforced lithium silicate CAD/CAM ceramic material.

## 2. Materials and Methods

### 2.1. Specimen Preparation

CAD/CAM blocks of a zirconia-reinforced lithium silicate ceramic material (Suprinity, Vita Zahnfabrik H. Rauter GmbH& Co., Bad Säckingen, Germany; Lot No. 48521) were used in this study. The typical composition of this material, described by the manufacturer, is the following: SiO_2_: 56–64%, Li_2_O: 15–21%, K_2_O: 1–4%, P_2_O_5_: 3–8%, Al_2_O_3_: 1–4%, ZrO_2_: 8–12%, CeO_2_: 0–4%, La_2_O_3_: 0.1%, Pigments: 0–6%.

Samples were fabricated by cutting, from the blocks, 100 bars (4 ± 0.2 mm × 1.2 ± 0.2 mm × 14 ± 0.2 mm, according to ISO 6872 [52]), 75 plaques (5 ± 0.2 mm × 4 ± 0.2 mm × 2 ± 0.2 mm) and 25 cubes (4 ± 0.2 mm × 4 ± 0.2 mm × 4 ± 0.2 mm), with the aid of a diamond saw and a precision cutting machine under constant water cooling (Isomet 1000, Buehler, Lake Bluff, IL, USA). The samples were calibrated and dimensionally controlled using a digital caliper (CALDI-6 MP, Truper, Shanghai, China). Sample surfaces were then standardized using SiC 600# grit sandpaper to simulate CAD/CAM machining process and homogenize all specimens’ roughness baseline. Crystallization of samples was carried out following the manufacturer’s firing parameters (starting temperature/drying time: 400 °C/4 min, heating time: 8 min, temperature rise rate: 55 °C/min until 840 °C for a holding time of 8 min, vacuum holding time: 8 min and long-term cooling until 680 °C) using a multipurpose ceramic furnace (VIP Executive 500, Jelrus/Whip Mix, Louisville, KY, USA). After crystallization, the samples were dimensionally controlled, registered and stored for group division and further treatment.

**Figure 1:**
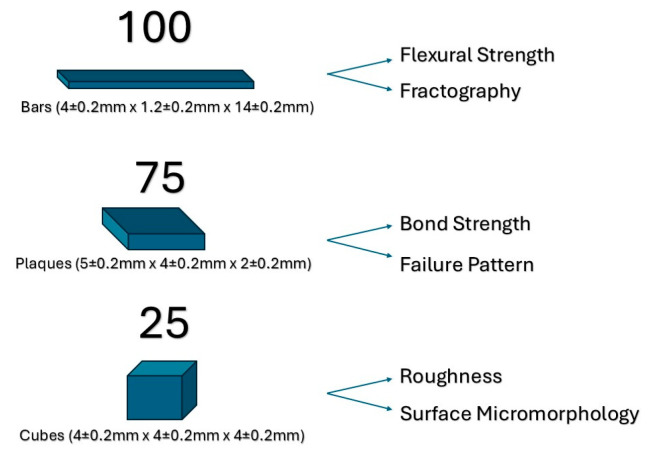
Schematic representation of all specimens prepared for this study.

Sample size determination was performed employing a power/sample size calculation test (Minitab v17.2.1, Minitab Inc.; State College, PA, USA) with a confidence interval of 95%. The standard deviation and expected confidence interval error, both necessary to perform this test, were collected from a pilot study and previous research (for numerical variables: flexural strength, bond strength, and roughness measurement). For the flexural strength test, the minimum significant sample size was 20 specimens, for bond strength, it was 15, and for roughness evaluation, it was 5 specimens. Following this analysis, sample sizes were defined as follows: flexural strength (bars), *n* = 20; bond strength (plaques), *n* = 15; and for roughness assessment (cubes), *n* = 5. A schematic representation of specimens’ shapes and distributions is displayed in Figure 1.

Bars, plaques, and cubes were equally distributed into five groups to be treated according to different etching protocols: 1. control: no other treatment than SiC paper regularization was performed; 2. HF5%20s: 5% hydrofluoric acid (Condac porcelana 5%, FGM, Joinville, Brazil; Lot No. 080217) was applied for 20 s; 3. HF5%60s: 5% HF was applied for 60 s; 4. HF10%20s: 10% HF (Condac porcelana 10%, FGM, Brazil; Lot No. 310717) was applied for 20 s; and 5. HF10%60s: 10% HF was applied for 60 s. According to the manufacturer’s instructions, all treatments were carried out, washing all specimens with air-powered water from the three-way syringe after the respective etching time elapsed. The specimens were also cleaned in an ultrasonic bath with distilled water for 5 min, dried, and preserved in a hermetically closed environment until the respective tests’ performance for each specimen. The plaques were additionally treated with a silane-MDP-containing ceramic primer (Monobond N, Ivoclar Vivadent, Schaan, Lichtenstein) for 60 s, air-dried for 30 s, and reserved for 3 min before being used. A single-blinded and previously calibrated operator performed sample preparation, surface treatment, and evaluation methods.

### 2.2. Roughness Assessment

Treated specimens (cubes) were submitted to surface roughness analysis using a contact profilometer (Dektak XT, Bruker, Billerica, MA, USA) employing a 0.5 µm tip. Five scanning lectures were performed, equally distributed through each central specimen area (3000 × 3000 µm) at a cross speed of 8.7 µm/sec and a resolution of 0.14 µm/pt.

The profile roughness mean (Pa) was selected to analyze the samples’ surface roughness, and an average was calculated from the five lectures performed to extract each specimen’s final measurement. Data were recorded and tabulated to be statistically analyzed.

### 2.3. Surface Micromorphology Evaluation

The same cubes used for roughness evaluation were also analyzed in a scanning electron microscope (SEM). They were sputter-coated with a gold-palladium powder alloy, placed on aluminum stubs with the aid of carbon adhesive tape, and taken into the microscope (JSM 5000, JEOL, Tokyo, Japan) operating at 15 kV and a working distance of 20 mm. Each group’s representative images were obtained at various magnifications (100×, 500×, 2000×, and 5000×) to analyze the surface micromorphology. For greater morphological detail, only 5000× magnification images are presented in this report.

### 2.4. Flexural Strength Analysis

Treated bars were submitted to the three-point bending test following all the indications described in the ISO 6872 [52]. It was performed using a universal testing machine (H10 KS, Tinius Olsen, PA, USA). A specific jig consisting of two support rollers was used, where the specimen lied (1.5 mm diameter, positioned 12 mm apart from their centers) and a third equal roller which applied the load (at the midpoint between the support rollers) at a crosshead speed of 1 mm/min. The flexural strength (σ) was calculated in MPa according to ISO 6872 equation: 3Pl/(2wb^2^), where P is the fracture load (N), l is the distance between the support rollers (mm), w is the specimens’ width, and b is the thickness of the specimen (mm). Data were recorded and tabulated to be statistically analyzed.

### 2.5. Fractographic Analysis

Fragments from the flexural strength experiment were collected and evaluated using a digital stereomicroscope (Jiusion Ltd., Shenzhen, China) using magnification ranging from 40x to 500x. Representatives from each group were chosen and prepared to be evaluated in SEM (JSM 5000, JEOL, Tokyo, Japan) using magnifications from 60× to 350× to determine the fracture origin and associated features. Specimens were prepared to employ the same procedure described in Section 2.3.

### 2.6. Microshear Bond Strength Evaluation (µSBS)

Seventy-five resin cement cylinders (Variolink Esthetic LC, Ivoclar Vivadent, Schaan, Lichtenstein) (1 ± 0.1 mm in diameter and 1 ± 0.1 mm in height) were built up (one cylinder was bonded to each ceramic plaque) on treated ceramic surfaces using a circled silicon mold (*n* = 15). Resin cement was injected onto the mold with the aid of an auto-mixing tip until the compartment was filled. A glass slide (0.5 mm thick) was placed over the top of the mold, and each cylinder was exposed for 20 s using an LED light-curing unit (Elipar Deep Cure, 3M, Saint Paul, MN, USA; light output: 1100 mW/cm^2^). The molds were removed after 5 min, and the specimens were water-rinsed, dried, and stored in relative humidity at 37 °C for 24 h. Afterward, each ceramic plaque was attached to a holding device previously adapted to a universal testing machine (Instron Electropuls E3000, Instron Corp, Canton, MA, USA) to perform microshear bond testing (µSBS), following the method developed by Shimada et al. [53]. A thin wire (0.2 mm) attached to the testing machine was placed surrounding the extreme base of each resin cement cylinder parallel for and in contact with the bonding area to apply an uplifting shear load at a crosshead speed of 0.5 mm/min. Bond strength was calculated considering the cross-sectional bonded area of each specimen and the load needed to separate both materials. All values were expressed in MPa.

### 2.7. Microshear Bond Strength Failure Pattern Evaluation

De-bonded areas of resin cement/ceramic plaques assemblies were analyzed after performing the bond strength experiment, using a digital stereomicroscope (Jiusion Ltd., China) using magnification ranging from 40× to 500×. Representatives from the most prevalent failure patterns were chosen and prepared to be evaluated in SEM (JSM 5000, JEOL, Tokyo, Japan) using magnifications from 50× to 60×. Specimens were prepared to employ the same procedure described in Section 2.3. Fractures were classified as adhesive failure between both materials (A), cohesive failure within the ceramic material (CC), cohesive failure within the resin cement layer (CR) or mixed (M) when a combination of more than one failure type occurred.

### 2.8. Statistical Analysis

Roughness means, bond strength and flexural strength data were submitted to the analysis of the parametric assumptions (normality by employing the Anderson–Darling test; and variance homogeneity by applying the Bartlett test) to evaluate the viability of performing such a statistical test. Afterwards, one-way ANOVA and Tukey’s post hoc tests were applied to all data sets (Minitab v17.2.1, Minitab Inc.; State College, PA, USA). Additionally (as described in the ISO 6872), the Weibull analysis was also executed with flexural strength data, calculating the Weibull modulus (m, numerically and graphically) and characteristic strength (σ0), both with their respective confidence intervals, using the maximum likelihood ratio (Minitab v17.2.1, Minitab Inc.; State College, PA, USA) [54]. All tests were performed using a confidence interval of 95%. Within the Weibull parameters, differences between groups were determined by rank order statistics, which means that if two groups presented any overlap between their confidence intervals, they were considered not statistically different from each other.

## 3. Results

### 3.1. Roughness Assessment

**Figure 2:**
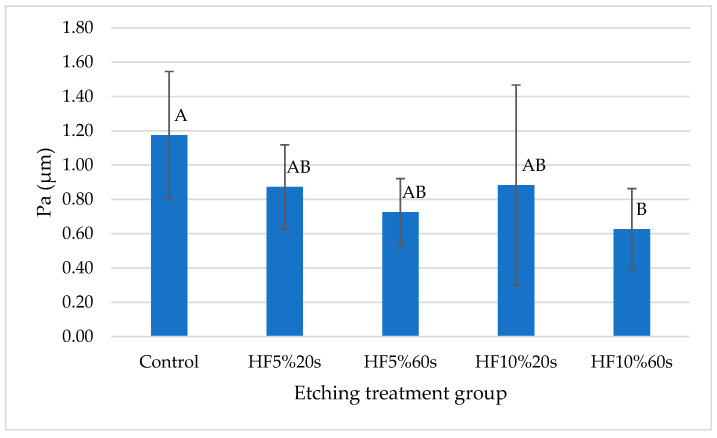
Graphical representation of Table 1 containing roughness mean values and standard deviations (expressed in MPa) from each group. Different letters represent significant statistical differences.

Roughness profile mean values (Pa) were not normally distributed (Anderson–Darling, *p* ˂ 0.05). Data were transformed by applying the Box–Cox procedure (λ = −0.331012) to fit them into a normal distribution (Anderson–Darling, *p* = 0.522). Homoscedasticity was also confirmed after this process (Bartlett, *p* = 0.425), allowing for a parametric statistical test, using one-way ANOVA. It revealed that the factor “etching protocol” was statistically significant for this variable (*p* = 0.003). According to Tukey’s post hoc test, the control group (C) showed the highest Pa value, not statistically different from groups using HF5% or HF10%20s, while group HF10%60s presented the lowest Pa means. However, this group did not differ from groups using HF5% and HF10%20s (Table 1, Figure 2).

### 3.2. Surface Micromorphology Evaluation

**Figure 3:**
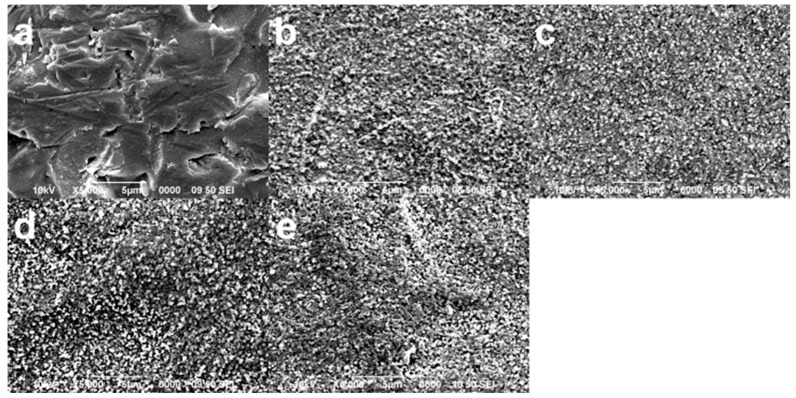
Surface micromorphology of zirconia-reinforced lithium silicate ceramic (ZLS) after being treated with the different etching protocols. SEM (Scanning Electron Microscope) images showing the typical surface morphology produced by the experimental groups: (**a**) control group (no etching); (**b**) HF5%20s group; (**c**) HF5%60s group; (**d**) HF10%20s group; and (**e**) HF10%60s group.

Morphological analysis revealed that the surface pattern is etching-protocol-dependent, as each treatment produced different characteristics within the material’s surface. The control group (no etching, only SiC sandpaper regularization prior firing) showed a very irregular surface where the glass-matrix exhibited some continuity disruptions such as scratches and voids (Figure 3a). The other groups presented a different morphology from the control group because the glass-matrix is not visible; instead, the crystalline phase shows many irregular-shaped tiny crystals (most of them below micron-scale) distributed along the whole surface. The HF5%60s etching protocol produced the most regular surface morphology (Figure 3c), while the most irregular seemed to be the surface treated under the HF10%20s protocol (Figure 3d), exhibiting more “dark areas” compared with the other groups, which may be interpreted as deeper depressions in SEM images. Both groups to which the etchant was applied for 60 s also presented those “dark areas”, these being darker (more profound defects) but more focused on the group using 10% HF than on the group employing 5%HF (Figure 3b,e).

### 3.3. Flexural Strength Analysis

**Figure 4:**
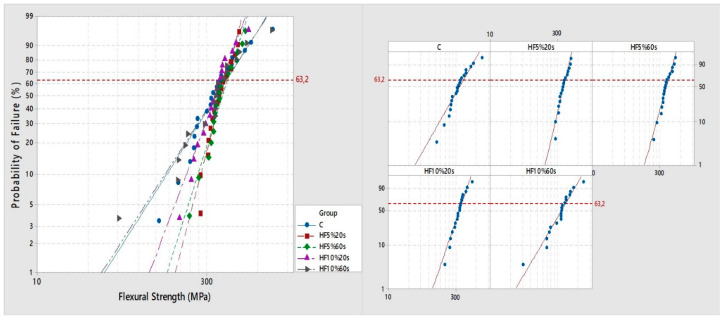
Weibull modulus plots for each group. The left plot shows the graphical Weibull modulus corresponding to all experimental groups (control: blue; HF5%20s: red; HF5%60s: green; HF10%20s: purple; HF10%60s: gray). Right side plots show a separate graphical representation for each groups’ Weibull modulus. All Weibull plots represent a curve relating the probability of failure % (*Y*-axis) and flexural strength in MPa (*X*-axis). The Weibull characteristic strength is pointed with the discontinuous red horizontal line, across a 63.2% probability of failure.

After confirming the normality (Anderson–Darling, *p* = 0.165) and homoscedasticity (Bartlett, *p* = 0.640) of the flexural strength data, ANOVA revealed that the “etching protocol” factor was not statistically significant (*p* = 0.473). Regarding the Weibull parameters, no significant differences were detected within the characteristic strength (σ0) among groups either; however, significant differences were noted within the Weibull modulus (m) for some groups (Table 2). Groups employing 5% HF obtained a significantly higher Weibull modulus than the HF10%60s and C groups, these last two not being statistically different from group HF10%20s (Table 2). Weibull modulus plots for all groups are displayed in Figure 4.

### 3.4. Fractographic Analysis

**Figure 5:**
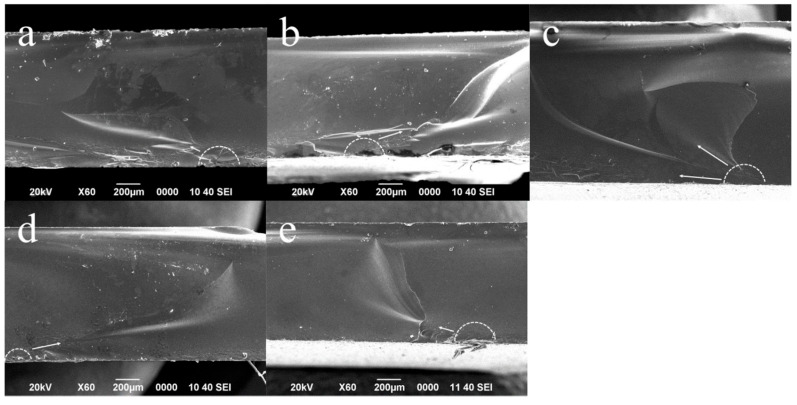
Representative SEM images (60×) from each group regarding fractographic analysis: (**a**) control group (no etching); (**b**) HF5%20s group; (**c**) HF5%60s group; (**d**) HF10%20s group; and (**e**) HF10%60s group. The discontinuous semicircle points to the origin of fracture for each group, which was located at surface defects on the treated side (tensile side), showing that the main direction of fracture propagation (arrows) to the compression side compression curl area is also notorious.

All analyzed fragments showed that fractures originated from a defect on the treated surface (tensile side, opposite to the side where the load was applied). All cracks propagated from defects within such a surface to the compression side, showing different features (Figure 5).

### 3.5. Microshear Bond Strength Evaluation (µSBS)

**Figure 6:**
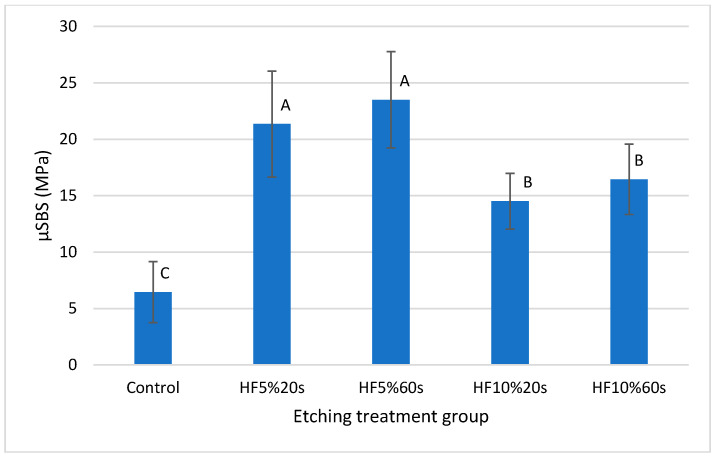
Graphical representation of Table 3 containing microshear bond strength mean values and standard deviations (expressed in MPa) from each group. Different letters represent significant statistical differences.

Normality (Anderson–Darling, *p* = 0.473) and homoscedasticity (Bartlett, *p* = 0.073) were confirmed. ANOVA revealed that the “etching protocol” factor was statistically significant (*p* ˂ 0.0001). Tukey’s test revealed that the groups treated with 5%HF resulted in higher µSBS compared to the groups treated with 10%HF, while all HF-treated groups showed higher µSBS mean values than the control group (Table 3 and Figure 6).

### 3.6. Microshear Bond Strength Failure Pattern Evaluation

**Figure 7:**
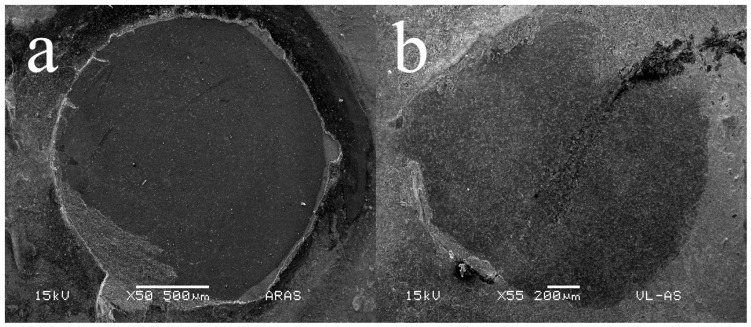
Representative SEM photo-micrographs of the most prevalent failure patterns obtained from microshear bond strength test: (**a**) adhesive failure, (**b**) mixed failure.

The most prevalent failure pattern for the control group was adhesive failure (A), while groups treated with HF showed a mixed failure pattern (M) mostly, this pattern being more prevalent on the 5%HF groups (Table 4 and Figure 7).

## 4. Discussion

Statistical analysis revealed that employing different HF etching protocols affected the surface roughness, bond strength, and Weibull modulus of zirconia-reinforced lithium silicate CAD/CAM ceramic; however, the etching protocol did not influence the material’s flexural strength (nor the Weibull characteristic strength). Therefore, the null hypothesis has to be partly rejected.

CAD/CAM materials in dentistry are constantly evolving, and this is the reason why research assessing the effectiveness of clinical procedures in recently developed materials is still relevant. Thus, ZLS ceramic was chosen as the main subject of this work. Additionally, its similarity to one of the most successful materials available nowadays (lithium disilicate) makes this material of great interest as an alternative to the first [3,4,5]. The surface treatments studied here were chosen because 4–5% and 9–10% are the most available concentrations of HF (commercially speaking), and 20 and 60 s are the most used application times within glass-ceramics [28,29,30,31]. To investigate bonding performance, µSBS was chosen due to its convenience in employing highly resistant materials such as CAD/CAM ceramics while still being an accurate and very used method [28,32,33]. Some issues of concern have been highlighted in tests employing shear. However, they can be minimized by taking some precautions such as those applied here. Roughness evaluation was performed to measure the effect of the chosen treatments on the material’s surface, relating it to SEM topographical evaluation and its mechanical properties to measure the extension of such effects on the structural behavior of this material. A three-point bending test was used due to its specimens’ design suitability with CAD/CAM blocks’ geometrical shape, also a widely validated method [4].

Regarding roughness, the current outcomes revealed that the roughest surfaces were recorded among the control group (no HF, only SiC sandpaper regularization) and the lowest within the HF10%60s group (Table 1 and Figure 2). SEM micromorphology analysis showed some sharper scratches in the control group, product of the regularization made to all specimens with SiC sandpaper. The procedure may explain the rise in the control group’s roughness, as reported previously for other glass-ceramics [47,49]. It is also likely and potentially dangerous because ZLS ceramic has been noted to be susceptible to developing residual crack growth when roughened in the pre-crystallized stage, as happens when milling a restoration [4,18].

HF acts by dissolving part of the SiO_2_ content on glass-ceramics as follows: SiO_2_ + 4HF→SiF_4_ + 2H_2_O/4SiF_4_ + 3H_2_O + 2HF→ 3H_2_SiF_6_ + H_2_SiO_3_. This will erode the surface of the ceramic, exposing the crystalline structures beneath the original glassy matrix [33,39]. It appears that besides dissolving part of the glassy matrix of this material, HF etching can also regularize its macrosurface morphology, disguising sharper defects, caused, in this case, by SiC sandpaper [4]. Surface roughness remained statistically comparable within groups employing 5%HF and HF10%20s group. These methods show a macro-homogenizer effect on materials’ surface morphology [45]. However, it is striking that, at least numerically speaking, the roughness was slightly higher when HF was applied for 20 s than for a longer time. It probably happened because 20 s may not be enough time for 10%HF to regularize this material’s surface macro-topography, leaving some sharper defects (Figure 3). In addition, 10% concentration applied during shorter periods may produce sharper, more profound, and localized defects within materials’ surface instead of producing a regular etching pattern within the horizontal extension of the surface [30]. This effect could be more significant if the material already shows previous defects or micro-scratches, as produced in the current work by the SiC sandpaper or in an actual situation by the milling bur [30].

On the other hand, when 10%HF was applied, it produced smoother surfaces. In this regard, it is essential to point out that ZLS is constituted by a conjunction of three phases comprising mostly spherical homogenous tiny crystals (~0.5 µm) [8]. Such composition may favor easier and greater crystal dislodgement when 10%HF is applied for a longer time to this material, producing a smoother and more stable macrosurface [3,4,15,19].

The microstructure and surface roughness of glass-ceramics are indeed not isolated factors. Both are related and could affect materials’ mechanical properties, and this is the reason why it is well documented that achieving smoother surfaces improves their mechanical behavior [20,35]. Additionally, a proper bonding between the ceramic material and the luting agent also influences the whole system’s mechanical behavior [29]. Following this rationale, flexural strength and bonding performance were analyzed when applying variations on HF etching parameters.

Several studies have investigated this relationship between etching treatments and mechanical properties, mainly evaluating flexural strength or fatigue failure load resistance within other glass-ceramics. Outcomes in this regard are controversial. Some authors have reported a significant negative effect of HF etching on materials’ mechanical behavior [7,36,38,39,50,51], while some others reported no significant influence of HF etching [37,40,41,42,44,47]. In contrast, fewer authors have determined a positive effect of HF etching on glass-ceramics mechanical properties [45,46,49]. Specifically, about ZLS, only a few of those previous works addressed this issue but with significant variability between study parameters and results. For example, Barchetta et al. and Monteiro et al. reported a positive effect of increased application time [45,49], while Hallman et al. found that the recommended etching protocol for this material (HF5%20s) reduced its flexural strength by around 20.9% compared to the control [38].

The current outcomes revealed that different HF etching protocols did not significantly affect the flexural strength of a ZLS CAD/CAM ceramic. However, significant differences were detected in the Weibull modulus (*m*) of some groups (Table 2, Figure 4). Weibull analysis is one of the most used tests to characterize ceramics’ mechanical behavior by associating a prediction of failure associated with two parameters. The characteristic strength or scale (*σ*_0_) is the strong point, where 63.2% of the specimens would have failed. And the Weibull modulus describes the variation in strength data (*m*, shape), related to a calculation of the distribution of defects or flaws within a materials’ microstructure to infer its mechanical consistency [54].

The flexural strength values and Weibull parameters obtained here are similar to those previously reported for this type of material (ZLS) [17,20,21,45,55]. The control and HF10%60s groups showed the lowest Weibull modulus. In the case of the control group, visible surface defects, a product of SiC sandpaper regularization (as previously discussed), are evident (Figure 3) and also consistent with the roughness results (Table 1). The greater prevalence of those flaws probably increased the variability in this group’s mechanical reaction to bending forces, making it less reliable than the 5%HF groups (Table 2, Figure 4). The lowest Weibull modulus produced by 10%HF may be explained by a possible, more substantial effect of this concentration, producing deeper and sharper defects within the surface 28,30. Such zones (Figure 3) may accumulate tension and develop a faster crack growth rate than smoother or more uniform regions within a surface [4,15,18,43]. It can also be supported by the fractographic analysis, which revealed that most of the fractures originated in surface defects within the treated side of the specimens (Figure 5).

Regarding the application time, both 5%HF groups showed a statistically comparable Weibull modulus, and roughness means. However, judging by the morphological analysis (Figure 3), it can be said that 60 s exerted a more homogenous macro-effect on the materials’ surface than 20 s did. HF in contact with the material’s surface for a short time probably produces more localized etching in vulnerable zones, such as previous defects (Figure 3). At the same time, a longer application time may etch materials’ surfaces more horizontally than vertically. A macro-smoother surface has been recommended in terms of mechanics for this specific material [3,4,5,15,18,49].

Bond strength was also significantly affected by HF etching protocols (Table 3). The control group showed poor bonding with resin cement compared to all treatments employing HF and silane as previously reported [23,26,32]. This result was consistent with the greater prevalence of the adhesive failure type recorded for this group (Table 4, Figure 7). It reaffirms the beneficial effect of promoting micromechanical and chemical interactions between ceramic materials and resin types of cement. The most prevalent failure type (mixed) obtained by HF-treated groups also reflects this interaction (Table 4, Figure 7). Groups employing 5%HF produced better bonding values than the 10%HF groups. This probably occurred because 10%HF may have produced deeper and sharper cone-shaped surface defects [30], hindering the resin cement from infiltrating them fully and thus affecting ceramic/cement bonding [34]. Analyzing failure patterns in bond strength testing is essential, as the debonded area provides critical information about the interaction between the bonded components and their behavior during debonding. The literature suggests that adhesive failures indicate poor interaction between the substrate and the adherent material, as no remnants of either part remain attached after separation [23,26,32]. Clinically, this could result in complete debonding of an indirect restoration. In contrast, mixed failures show areas where the adherent material remains attached to the substrate after separation, indicating an intimate interaction between the two materials in certain regions. These areas likely experienced cohesive failures within the adherent material (in this case, resin cement), meaning that the adhesive bond in those spots was stronger than the material’s cohesive strength. Clinically, this scenario is preferred to ensure long-term reliable bonding of the indirect restoration. While fully cohesive failures within the adherent material also suggest strong bonding, in microshear bond strength tests, they may indicate a potential bias in test execution. Specifically, such failures could suggest that the shear force applied during testing was not fully parallel to the bonded area. Therefore, a higher prevalence of mixed failure types, as observed in this study, is preferred over fully cohesive failures.

From previous outcomes, it can be said that the most suitable etching protocol for glass-ceramics is material-dependent. Moreover, no automatic extrapolation should be made from one type of glass-ceramic to another. That scenario would apply to materials belonging to the same sub-class, for example, lithium disilicate and lithium silicate-based ceramics. According to their glassy content and bond strength to resin-based materials, the previous tendency in the literature has been to recommend an etching time to treat glass-ceramics [28]. However, the rising concern of the effect of such treatments on the structural integrity and mechanical behavior of CAD/CAM materials has led to an integral approach [30,38]. Therefore, an etching treatment employing 5%HF (followed by a silane coupling agent) can be recommended to treat ZLS CAD/CAM ceramics based on the current results. This recommendation agrees with a recent study that determined that 4.9%HF produced better ceramic/cement bonding on this material, compared to 9.5%HF [23]. So, within the limitations of this in vitro study, this information may be considered clinically significant and should be taken into account when looking for proper surface treatment for this material.

Some limitations of this in vitro study are the use of a static mechanical test instead of a dynamic method, a test set-up directed to simulate only the pre-cementation performance of the tested material instead of reproducing a methodological approach directed to emulate the clinical–mechanical performance of this material in long-term service, and a lack of comparison between ZLS and other similar materials, such as lithium disilicate ceramics. However, the aim of this specific work was successfully achieved, and these outcomes can be considered a starting point for further investigations in this regard. Future research could address these aspects by evaluating the influence of these etching protocols on the fatigue resistance and lifetime predictions of cemented restorations made from this material, as well as comparing them to lithium disilicate restorations.

## 5. Conclusions

Within the limitations of this in vitro study and based on the current results, it can be concluded that variations in hydrofluoric acid (HF) etching protocols do not influence the ZLS CAD/CAM ceramics’ flexural strength; however, those variations do affect its surface roughness, bond strength, and Weibull modulus. HF etching is necessary to create a rough surface topography on this ceramic to improve interlocking with the luting material and regularize some previous sharp surface defects. The best overall outcomes were achieved when using 5%HF to treat this material.

## Figures and Tables

**Table 1:** Roughness profile mean values and standard deviations (Pa) recorded for each etching protocol group.

Etching Protocol	Pa (µm)
Control	1.176 (0.370) A
HF5%20s	0.873 (0.245) AB
HF5%60s	0.726 (0.195) AB
HF10%20s	0.884 (0.583) AB
HF10%60s	0.627 (0.236) B

Different letters mean statistical differences among groups (Tukey, *p* < 0.05).

**Table 2:** Flexural strength (*σ*) in MPa with the respective standard deviation, Weibull modulus (*m*) and characteristic strength in MPa (*σ*_0_) with their respective confidence intervals for all groups.

Group	σ	(*m*)	(σ_0_)
Control	312.24 (29.12)	2.78 (2.04–3.79) B	330.43 (310.35–354.16)
HF5%20s	316.38 (22.25)	6.70 (4.65–9.65) A	329.14 (319.79–339.22)
HF5%60s	321.89 (22.81)	5.63 (3.97–7.99) A	332.27 (321.27–344.28)
HF10%20s	314.08 (24.74)	4.69 (3.39–6.48) AB	322.53 (310.68–335.65)
HF10%60s	308.58 (20.99)	2.73 (1.96–3.79) B	329.53 (308.82–354.18)

Different letters mean statistical differences among groups (Tukey, *p* < 0.05).

**Table 3:** Microshear bond strength mean values and standard deviations (expressed in MPa) correspond to each group.

Etching Protocol	µSBS (MPa)
Control	6.46 (2.71) C
HF5%20s	21.35 (4.70) A
HF5%60s	23.50 (4.27) A
HF10%20s	14.51 (2.47) B
HF10%60s	16.45 (3.12) B

Different letters mean statistical differences among groups (Tukey, *p* < 0.05).

**Table 4:** Failure type prevalence (%) of de-bonded specimens for each group.

	Failure Type Prevalence (%)			
Etching Protocol	M	CC	CR	A
Control	20	0	0	80
HF5%20s	87	0	0	13
HF5%60s	100	0	0	0
HF10%20s	73	0	0	27
HF10%60s	60	5	0	35

## Data Availability

The original contributions presented in the study are included in the article, further inquiries can be directed to the corresponding author.
